# Recurrent Syncope in a Patient With Arrhythmogenic Right Ventricular Cardiomyopathy

**DOI:** 10.7759/cureus.45850

**Published:** 2023-09-24

**Authors:** Efrain Castillo

**Affiliations:** 1 Medicine, Universidad Latina de Panama, Panama City, PAN

**Keywords:** arrhythmias, arrhythmogenic right ventricular dysplasia, cardiomyopathies, recurrent syncope, epsilon wave

## Abstract

Arrhythmogenic right ventricular cardiomyopathy (ARVC) is an autosomal inherited cardiac condition characterized by fibroadipose tissue replacement of the right ventricular muscle, leading to structural changes and a high risk for ventricular arrhythmias, a gradual decline in right ventricular function, and sudden cardiac death. ARVC has an autosomal dominant inheritance pattern with variable expression among patients, typically affecting young adults. Genetic mutations affecting the cardiac desmosome genes have been widely reported. Intense exercise has been hypothesized as one of the drivers of ARVC's pathogenesis. Due to its non-specific presentation, it can become a diagnostic challenge for physicians with delayed care. We report a case of a male adult with a history of recurrent syncope and atypical chest pain who developed ventricular tachycardia on admission. This case aims to highlight the unspecific manifestations of ARVC and its main electrocardiographic features for an early diagnosis.

## Introduction

Arrhythmogenic right ventricular cardiomyopathy (ARVC) is a phenotype that is part of the arrhythmogenic cardiomyopathy spectrum (ACM). ARVC is an autosomal dominant hereditary disease characterized histologically by the replacement of cardiomyocytes with fibrofatty tissue. Patients with ARVC are predisposed to cardiac chamber enlargement and have an increased risk for ventricular arrhythmias, right ventricular failure, and sudden cardiac death (SCD) [1]. The incidence of ARVC is unknown, but there is an estimated prevalence from 1 in 2000 to 1 in 5000 in the general population [2]. Initial descriptions of ARVC focused on the arrhythmic substrate of some areas of the right ventricle (RV), specifically the subtricuspid RV wall, the RV apex, and the RV outflow tract [3] with recent studies supporting the presence of a new biventricular triangle, adding the left ventricular (LV) posterolateral wall as part of the pathogenesis [4]. Currently, efforts have been made to improve our understanding, proposing new measures for early detection and genetic testing in high-risk patients. The pathophysiology has been broadened to include diffuse RV manifestations and single LV or biventricular involvement in the dilated phase, often indistinguishable from dilated cardiomyopathy [5]. The main goal of treatment is to reduce the risk of SCD and improve the quality of life of patients affected. This article aims to highlight the importance of recognizing the classical electrocardiographic features of ARVC. 

## Case presentation

**Figure 1:**
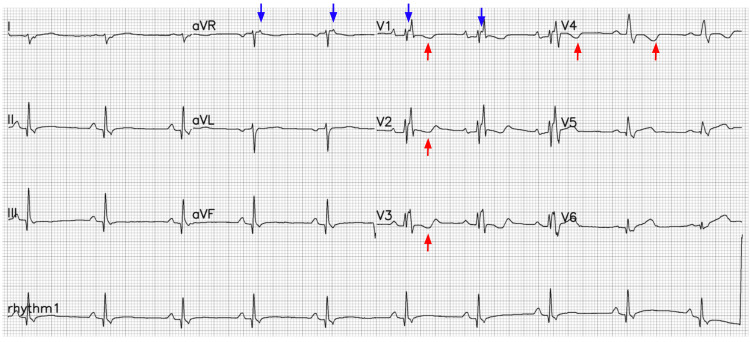
Electrocardiogram showing Epsilon wave in lead aVR and V1 (blue arrows) and prominent T wave inversions in V1-V4 (red arrows).

A 45-year-old Hispanic male patient presented to the ER after experiencing four episodes of syncope, accompanied by 12 hours of atypical precordial chest pain, palpitations, and vomiting. In the initial evaluation, he was identified as having monomorphic ventricular tachycardia. He was treated with synchronized cardioversion, amiodarone, and a bolus of propranolol. In the post-arrhythmic event, a 12-lead electrocardiogram showed T wave inversion in leads V1 to V4, right bundle branch block, and the presence of an Epsilon wave in aVR and V1 (Figure 1).

Upon admission, there were no histories or findings of hypertension or diabetes mellitus. The patient's medical history did include occasional use of nonsteroidal anti-inflammatory drugs (NSAIDs). He denied the use of other medications and the consumption of tobacco or illicit drugs but reported a 15-year history of alcohol use. Of note in the family history is his father's death at age 24 due to SCD. The system review revealed positive findings for lower limb edema, mild dyspnea, and non-radiating chest pain with an intensity of 7/10 but was negative for headache, fever, or adrenergic symptoms. A transthoracic echocardiogram showed a moderately dilated RV, and the cardiac MRI revealed an RV ejection fraction of 40%, regional RV dyskinesia, and RV fatty infiltration. The diagnosis of ARVC was established. Treatment with metoprolol was started, and the placement of an implantable cardioverter defibrillator was planned. Lifestyle recommendations were provided, including avoidance of strenuous physical activity, regular checkups, and cardiac evaluation for first-degree relatives. Supervised cardiac rehabilitation was offered once daily, five days a week, without arrhythmia recurrence or any other adverse effect. At the six-month evaluation, the patient reports improvement in symptoms and denies new episodes of syncope.

## Discussion

**Figure 2:**
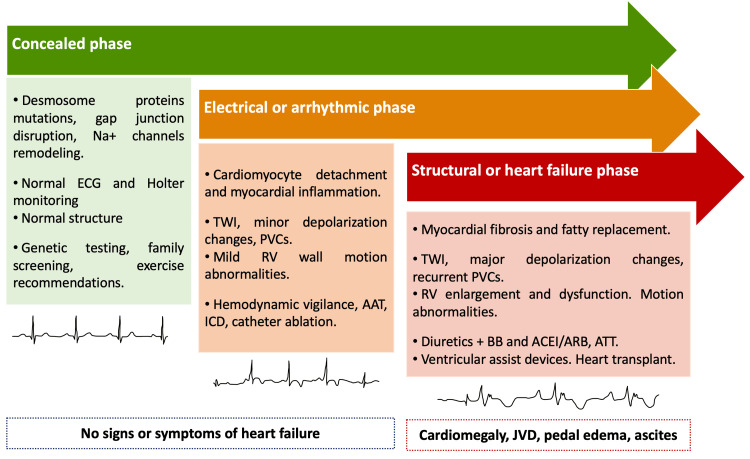
Clinical course of patients with ARVC. Na+: Sodium; ECG: Electrocardiogram; TWI: T-wave Inversion; PVC: Premature Ventricular Contractions; RV: Right Ventricle; AAT: Antiarrhythmic Therapy; ICD: Implantable Cardioverter-Defibrillator; BB: Beta-Blockers; ACEI: Angiotensin-Converting Enzyme Inhibitors; ARB: Angiotensin Receptor Blockers; ATT: Antithrombotic Therapy; JVD: Jugular Venous Distention; ARVC: Arrhythmogenic Right Ventricular Cardiomyopathy. Image credit: Efrain Castillo.

ARVC is typically considered an autosomal dominant hereditary disease with reduced penetrance and is associated with ventricular tachyarrhythmias, SCD, and heart failure [6]. Abnormal cardiac desmosomes, such as plakophilin-2 (PKP2), desmoglein-2 (DSG2), desmoplakin (DSP), and plakoglobin (JUP), drive the pathogenesis, progressively leading to cardiomyocyte necrosis, fibrofatty tissue replacement, and ventricular dilation [7]. Although RV involvement is more common, LV or biventricular disease can occur in up to 24% of cases, and LV dysfunction is observed in approximately 47% to 60% of cases [8]. The prevalence of ARVC is around 1 in 4,000 individuals [9]. This condition predominantly affects young adults, with a median age of 40 years, and stands as the second most common cause of SCD in individuals under 40 years [10]. Many causes have been proposed, including modifier genes effect, post-translational defects, epigenetic factors, and environmental effects such as young age, male sex, athletic training, and pregnancy. Desmosome-specific gene mutations are important contributors to cell-cell adhesion disruption and cardiomyocyte detachment since desmosomes are essential for myocyte cellular stability. Intercellular junctions are unable to adapt to any mechanical stress [11]. Some patients remain asymptomatic, while those with moderate to severe disease may exhibit symptoms such as dyspnea, palpitations, syncope, angina, edema, lightheadedness, and might even experience sudden death (Figure 2) [12]. Clinical manifestations typically emerge between adolescence and mid-adulthood. Currently, no single gold standard diagnostic test exists for ARVC, but diagnostic criteria have been established by the 2010 revised International Task Force and by the 2020 International criteria, also known as the 'Padua Criteria' (Table 1) [13]. The 2020 Padua Criteria broadens the spectrum of recognized ARVC phenotypes, accounting for previously underrecognized patient presentations that diverge from the original ARVC phenotype and introduces the term ACM to include LV and biventricular involvement.

**Table 1:** 2020 International Criteria for Diagnosis of ARVC (Padua Criteria). The definitive diagnosis of ARVC is established with at least one criterion either major or minor from category I or II fulfilled. ACM: Arrhythmogenic Cardiomyopathy; ALVC: Arrhythmogenic Left Ventricular Cardiomyopathy; CMR: Cardiac Magnetic Resonance; EDV: End Diastolic Volume; EF: Ejection Fraction; LBBB: Left Bundle‐Branch Block; LGE: Late Gadolinium Enhancement; LV: Left Ventricle; RBBB: Right Bundle‐Branch Block. *Global LV systolic dysfunction: EF <55% on echocardiography and <57% (nonathletes) or <58% (athletes) on cine CMR.

I. Global or regional dysfunction and structural alterations	
Major	Regional RV akinesia, dyskinesia, or bulging + ≥1 of the following: global RV dilatation or global RV systolic dysfunction.
Minor	Regional RV akinesia, dyskinesia, or aneurysm of RV free wall.
II. Tissue characterization of walls	
Major	By CE‐CMR: transmural LGE (stria pattern) of ≥1 RV region (inlet, outlet, and apex in 2 orthogonal views). By EMB: fibrous replacement of the myocardium in ≥1 sample, with or without fatty tissue.
III. Repolarization abnormalities	
Major	Inverted T waves in right precordial leads (V1-V3) or beyond in individuals with complete pubertal development and in the absence of complete RBBB.
Minor	Inverted T waves in leads V1-V2 in individuals with completed pubertal development in the absence of complete RBBB. Inverted T waves in V1-V4 in individuals with completed pubertal development in the presence of complete RBBB
IV. Depolarization/conduction abnormalities	
Minor	Epsilon wave in the right precordial leads (V1-V3). Terminal activation duration of QRS ≥55 ms measured from the nadir of the S wave to the end of the QRS, including R’, in V1, V2, or V3 in the absence of complete RBBB.
V. Arrhythmias	
Major	Frequent ventricular extrasystoles (>500/24 h), non‐sustained or sustained ventricular tachycardia of LBBB morphology*
Minor	Frequent ventricular extrasystoles (>500/24 h), non‐sustained or sustained ventricular tachycardia of LBBB morphology with inferior axis (“RVOT pattern”).
VI. Family history/genetics	
Major	ACM confirmed in a first‐degree relative who meets diagnostic criteria, confirmed pathologically at autopsy or surgery, or identification of a pathogenic or likely pathogenetic ACM mutation in the patient under evaluation.
Minor	History of ACM in a first‐degree relative in whom it is not possible or practical to determine whether the family member meets diagnostic criteria OR Premature sudden death (<35 years of age) due to suspected ACM in a first‐degree relative OR ACM confirmed pathologically or by diagnostic criteria in a second‐degree relative.

**Figure 3:**
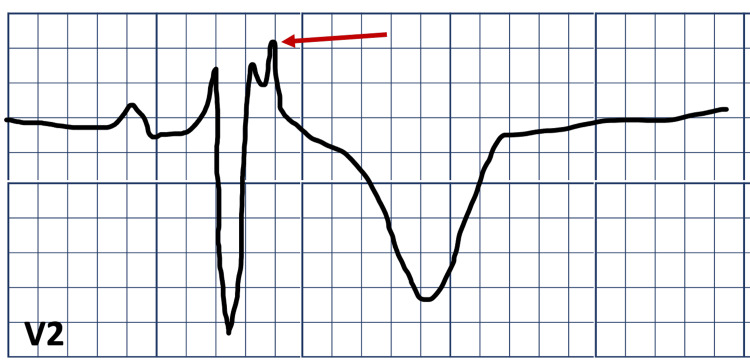
Epsilon wave in V2, described as a small deflection buried in the QRS complex, due to right ventricular delay. Image credit: Efrain Castillo.

The main electrocardiographic findings are repolarization or depolarization abnormalities. Most patients have T wave inversion on precordial leads V1-V3 in the absence of a right bundle branch block. Other findings are widened QRS, ventricular tachycardia, ventricular extrasystoles, and the presence of Epsilon waves [14]. The latter are low-amplitude electrical potentials hidden at the end of a QRS complex. The presence of the Epsilon wave (Figure 3) is one of the major criteria for diagnosis. It is the most specific electrocardiographic finding and is present in 30% of patients [15]. Endomyocardial biopsies confirm the diagnosis of ARVC but are rarely performed.

**Figure 4:**
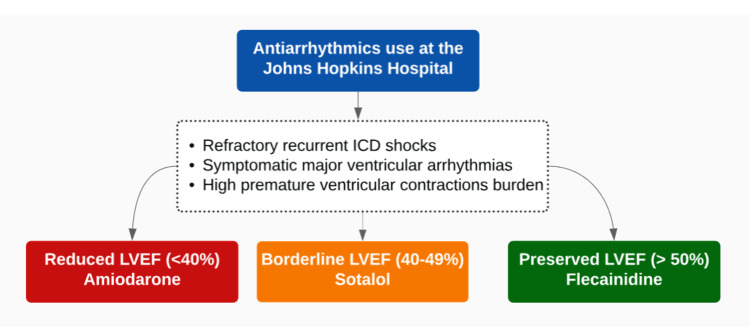
Antiarrhythmic drug indications for ARVC patients at the Johns Hopkins Hospital. ARVC: Arrhythmogenic Right Ventricular Cardiomyopathy. Image credit: Efrain Castillo.

The treatment of ARVC involves several key aspects, including evaluating the potential for sustained ventricular arrhythmia, assessing the need for an implantable cardioverter-defibrillator (ICD), and preventing malignant ventricular dysrhythmias and ICD interventions. Since there is a lack of high-quality evidence on the goal standard treatment for patients with ARVC, the Johns Hopkins Hospital has shown clinical excellence on this topic and has provided clinical guidelines for medical providers [16]. These goals can be achieved by the early use of amiodarone, class 1c antiarrhythmics, flecainide in particular, or beta-blockers such as sotalol in particular patients (Figure 4) [17], high-intensity exercise restriction, and catheter ablation. Antiarrhythmic agents may be needed in recurrent cases. An ICD is indicated in patients who have experienced ≥1 episode of hemodynamically unstable, sustained ventricular tachycardia or ventricular fibrillation [18]. It is also essential to consider cardiac screening for first-degree family members.

## Conclusions

In the early stages of ARVC, the pathogenesis remains concealed, often manifesting as depolarization or repolarization abnormalities without detectable clinical or cardiac imaging signs. The condition starts with desmosomal protein mutations, cell-cell electrical uncoupling, and disruption with progressive myocardial inflammation, fibrosis, and fibrofatty replacement. These pathological hallmarks increase the risk of ventricular dilation, RV wall motion abnormalities, and eventual ECG changes. Emphasizing the importance of electrical abnormalities over structural changes is crucial for diagnosing suspected ARVC. Epsilon waves are highly specific ECG findings and correlate with disease extent, ventricular dysrhythmias risk, and potential SCD risk. However, defining Epsilon waves remains challenging, necessitating more accurate detection methods. This case report highlights the importance of recognizing the most common electrocardiographic findings seen in patients with ARVC, including T wave inversion in precordial leads, Epsilon waves, and VT/VF. Diagnosis confirmation is critical to conducting family screening tests, preventing lethal arrhythmias and disease progression by treating patients with antiarrhythmic drugs and exercise recommendations, and assessing the need for other interventions.
